# Chronic Kidney Disease or Hypertension After Childhood Cancer

**DOI:** 10.1001/jamanetworkopen.2025.8199

**Published:** 2025-05-19

**Authors:** Asaf Lebel, Rahul Chanchlani, Vedran Cockovski, Allison Dart, Adam James Fleming, Amit X. Garg, Nivethika Jeyakumar, Kirby Kim, Abhijat Kitchlu, Eric McArthur, Danielle Nash, Paul C. Nathan, Rulan S. Parekh, Rachel Pearl, Jason Pole, Raveena Ramphal, Jennifer Reid, Tal Schechter-Finkelstein, Lillian Sung, Ron Wald, Stella Wang, Peter Wong, Michael Zappitelli

**Affiliations:** 1 Division of Nephrology, Department of Paediatrics, The Hospital for Sick Children, Toronto, Ontario, Canada; 2 Pediatric Nephrology Unit, Ha’Emek Medical Center, Afula, Israel; 3 Division of Pediatric Nephrology, Department of Pediatrics, McMaster University, Hamilton, Ontario, Canada; 4 Department of Pediatrics and Child Health, University of Manitoba, Winnipeg, Manitoba, Canada; 5 Department of Pediatric Hematology and Oncology, McMaster Children’s Hospital, Hamilton, Ontario, Canada; 6 London Health Sciences Centre, Lawson Health Research Institute, London, Ontario, Canada; 7 Patient Partner, The Hospital for Sick Children, Toronto, Ontario, Canada; 8 Division of Nephrology, Department of Medicine, University of Toronto, Toronto, Ontario, Canada; 9 ICES, Toronto, Ontario, Canada; 10 Division of Haematology and Oncology, Department of Paediatrics, The Hospital for Sick Children, Toronto, Ontario, Canada; 11 Pediatric Oncology Group of Ontario, Toronto, Ontario, Canada; 12 Division of Hematology and Oncology, Department of Pediatrics, Children’s Hospital of Eastern Ontario-Ottawa Children’s Treatment Centre, Ottawa, Ontario, Canada; 13 Division of Nephrology, St Michael’s Hospital and University of Toronto, Toronto, Ontario, Canada; 14 William Osler Health System, Brampton, Ontario, Canada

## Abstract

**Importance:**

Post–cancer therapy kidney outcomes, including chronic kidney disease (CKD) and hypertension, are common in childhood cancer survivors (CCS). The incidence and timing of CKD and hypertension in CCS compared with other at-risk or general populations are unclear.

**Objective:**

To determine the association of childhood cancer treatment with post–cancer therapy CKD or hypertension.

**Design, Setting, and Participants:**

Population-based matched cohort study of children treated for cancer between April 1993 and March 2020 in Ontario, Canada, with follow-up until March 2021. The CCS (exposed) cohort included children (≤18 years) surviving cancer. Comparator cohorts were a hospitalization cohort (children who were hospitalized) and a general pediatric population (GP) cohort (all other Ontario children). Exclusion criteria were history of previous cancer, organ transplant, CKD, dialysis, or hypertension. Matching with each of the 2 comparator cohorts was performed separately and in a 1:4 ratio by age, sex, rural vs urban status, income quintile, index year, and presence of previous hospitalization. Data were analyzed from March 2021 to August 2024.

**Exposure:**

Treatment for cancer.

**Main Outcomes and Measures:**

The primary outcome was the composite of CKD or hypertension, defined by administrative health care diagnosis and procedure codes. Fine and Gray subdistribution hazard modeling, accounting for competing risks (death and new cancer diagnosis or relapse) and adjusting for cardiac disease, liver disease, and diabetes, was used to determine the association of cancer treatment with outcomes.

**Results:**

There were 10 182 CCS (median [IQR] age at diagnosis, 7 [3-13] years; 5529 male [54.3%]; median [IQR] follow-up time, 8 [2-15] years) matched to 40 728 hospitalization cohort patients (median [IQR] age at diagnosis, 7 [2-12] years; 5529 male [weighted percentage, 54.3%]; median [IQR] follow-up time, 11 [6-18] years) and 8849 CCS (median [IQR] age at diagnosis, 5 [2-11] years; 4825 male [54.5%]; median [IQR] follow-up time, 7 [2-14] years) matched to 35 307 GP cohort individuals (median [IQR] age at diagnosis, 6 [2-11] years; 4825 male [weighted percentage, 54.5%]; median [IQR] follow-up time, 10 [5-16] years). Most frequent cancer types were leukemia (2948 patients [29.0%]), central nervous system neoplasms (2123 patients [20.9%]), and lymphoma (1583 patients [15.5%]). During observation, cumulative incidence of CKD or hypertension was 20.85% (95% CI, 18.75%-23.02%) in the CCS cohort vs 16.47% (95% CI, 15.21%-17.77%) in the hospitalization cohort and 19.24% (95% CI, 15.99%-22.73%) in the CCS cohort vs 8.05% (95% CI, 6.76%-9.49%) in the GP cohort. CCS were at increased risk of CKD or hypertension compared with the hospitalization cohort (adjusted hazard ratio, 2.00; 95% CI, 1.86-2.14; *P* < .001) and the GP cohort (adjusted hazard ratio, 4.71; 95% CI, 4.27-5.19; *P* < .001).

**Conclusions and Relevance:**

In this population-based study, CCS were at increased risk for CKD and hypertension, which are associated with mortality, suggesting that early detection and treatment of these conditions in CCS may decrease late complications and mortality.

## Key Points

**Question:**

What is the risk of chronic kidney disease (CKD) or hypertension over time among childhood cancer survivors (CCS)?

**Findings:**

In this retrospective matched cohort study over a 27-year period comparing 10 182 CCS with 40 728 children who had been hospitalized and a general pediatric population of 35 307 individuals, CCS had a significantly higher estimated risk of developing CKD or hypertension compared with those in the 2 comparison cohorts.

**Meaning:**

These findings suggest that the development of updated evidence-based international guidelines for screening of late kidney outcomes in CCS is warranted.

## Teaser

This cohort study analyzes associations of cancer treatment with later development of chronic kidney disease or hypertension among childhood cancer survivors in Canada.

## Introduction

Improved treatments and survival for childhood cancer^1,2,3^ have come with a cost of adverse cancer treatment effects; more than 99% of childhood cancer survivors (CCS) have chronic health problems by age 50 years.^4,5^ Long-term kidney outcomes, including chronic kidney disease (CKD) and hypertension, are common in CCS attaining adulthood.^6,7,8,9,10,11,12^ Childhood CKD is associated with many organ complications; even mild CKD is associated with early signs of cardiovascular disease (CVD).^13,14,15,16,17^ Hypertension is an important childhood cardiovascular risk factor and predicts adult hypertension, CVD, and mortality.^18,19,20,21^ In adults, CKD and hypertension are CVD and mortality risk factors,^22,23,24,25^ which is concerning for CCS who are at a known high risk for metabolic syndrome, hypertension, and CVD.^4,26,27,28,29^ Early treatment of CKD and hypertension slows progression and reduces complications.^24,30,31,32^

Incidence and timing of CKD and hypertension in CCS vs other hospitalization populations or the general population are unclear. Studies on CCS kidney outcomes have been relatively small or without comparator cohorts, which are acknowledged as major barriers to quantifying long-term kidney health risk.^6,8,9,10,33,34,35,36,37,38^ This lack of robust evidence may partially explain why CCS kidney health follow-up guidelines are vague and nonactionable.^39,40^ Administrative health care data research may help overcome this issue and provide evidence needed to improve follow-up guidelines.^41,42,43,44,45,46,47^ Using administrative health care data from Ontario, Canada, we determined the extent to which CCS are at higher risk for CKD or hypertension up to 27 years after cancer treatment, compared with 2 matched cohorts: a cohort of children who have been hospitalized and a healthy general pediatric population (GP) cohort.

## Methods

### Design and Setting

We performed a population-based retrospective matched cohort study of CCS who received a cancer diagnosis between April 1, 1993, and March 31, 2020, in Ontario, Canada (prevalent pediatric population approximately 3 million).^48^ Health care services in Ontario are publicly funded and universal. This project was authorized under Section 45.1 of the Ontario Personal Health Information Protection Act. Reporting in this study was consistent with the Reporting of Studies Conducted Using Observational Routinely Collected Health Data (RECORD)^49^ and the Strengthening the Reporting of Observational Studies in Epidemiology (STROBE)^50^ guidelines.

### Study Population

**Figure 1.**
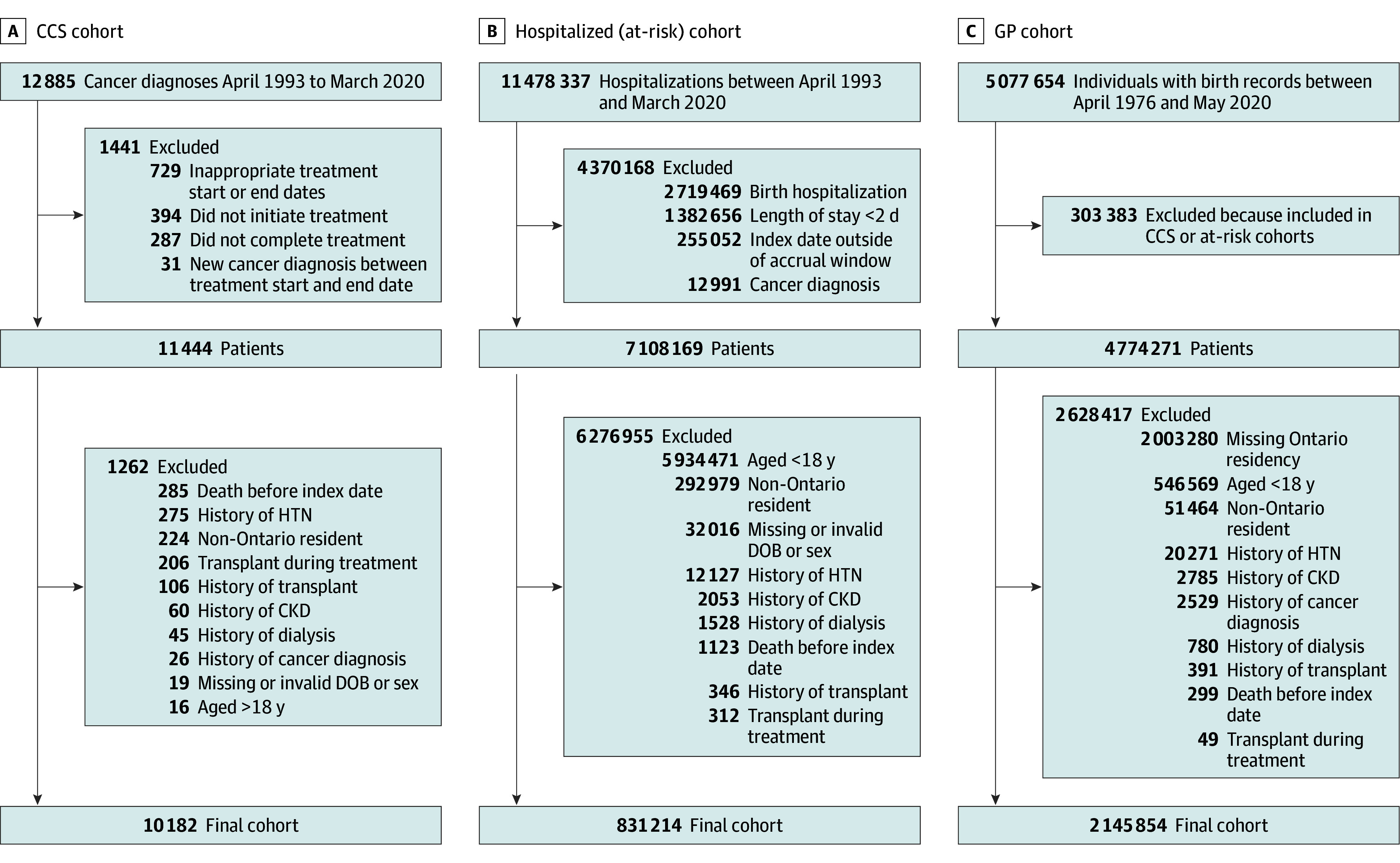
Study Flow This figure describes the initial population numbers in Ontario, Canada, during the study period for the population of interest (childhood cancer survivors [CCS]) and the 2 comparator cohorts (the at-risk or hospitalization cohort, consisting of children who have been hospitalized, and the general pediatric population (GP) cohort. Reasons for exclusion and numbers excluded are shown, leading to the final, unmatched analysis populations. CKD indicates chronic kidney disease; DOB, date of birth; HTN, hypertension.

**Figure 2.**
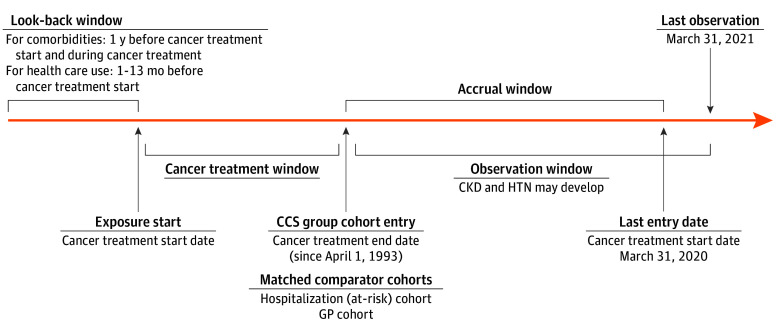
Study Observation Windows and Timelines The figure is focused on the time windows and observation period for the childhood cancer survivor (CCS) cohort, showing the look-back window (pre–cancer treatment), the cancer treatment window (from cancer treatment start to end), the index date, which is the date of cancer treatment end and also the start of follow-up, and the total observation period, which ranges from 1 to approximately 27 years. CKD indicates chronic kidney disease; GP, general pediatric population; HTN, hypertension.

The CCS (exposed) cohort included children who were aged 18 years or younger at diagnosis of a first cancer during the study period, who received cancer treatment (ie, not only observed or palliative), were alive at cancer treatment end (date of the last related cancer treatment), and were registered in the Pediatric Oncology Group of Ontario Network Information System (POGONIS) database, which includes children treated for cancer at the 5 Ontario pediatric cancer centers. Data were collected by trained personnel and are linkable to administrative health care data; POGONIS captures 98% of Ontario children younger than 15 years and 50% of children aged 15 years or older (Figure 1). Two comparator cohorts were selected. The hospitalization cohort included children 18 years or younger surviving non–birth-associated hospitalization of at least 2 days during the study period who did not have a cancer diagnosis. The GP cohort included children aged 18 years or younger registered in the Ontario Registered Persons Database^51,52,53^ (eTable 1 in Supplement 1) and not in the aforementioned cohorts (Figure 1). The CCS cohort follow-up start (index date) was cancer treatment end date (ie, alive at treatment end) (Figure 2). In the hospitalization cohort, the equivalent to treatment start date was hospital discharge date (ie, exposure just occurred). A comparable index date (ie, treatment end) and date comparable with cancer diagnosis in the CCS cohort was randomly assigned to individuals using a bootstrap approach based on duration between CCS treatment start and end date. For the GP cohort, a similar approach was used for all 3 pertinent dates, randomly assigning dates based on CCS cohort data distribution. Follow-up terminated on March 31, 2021, or outcome occurrence, death, new cancer diagnosis or relapse, emigration out of Ontario, or when time since last health care encounter exceeded 3 years. We excluded individuals with an invalid or missing date of birth or sex, non-Ontario residents, those aged older than 18 years at treatment start, those who died before the index date, those with a history of previous cancer or solid organ transplant, and patients with pre–cancer therapy (or equivalent in comparators) CKD, dialysis, or hypertension. We excluded CCS if they had a new cancer diagnosis or relapse between treatment start and end dates (Figure 1). We matched comparator cohorts to the CCS cohort using a 1:4 ratio (to balance maximizing power, minimizing bias and loss of CCS cohort patients)^54,55^ based on several sociodemographic variables (age at cohort entry [±90 days if ≤12 months or ±12 months if >12 months], sex, rural status [community <10 000 persons], neighborhood income quintile, and index year [±3 years]) and presence or absence of hospital admissions 1 to 13 months before cohort entry (avoiding inclusion of cancer diagnosis-related hospitalizations) to estimate baseline health care utilization. These matching variables allowed retention of matched sets within planned subgroup analyses.

### Data Sources

We utilized data from multiple linked provincial health administrative databases housed at ICES (formerly, Institute for Clinical Evaluative Sciences) in Ontario, Canada. eTable 1 and eTable 2 in Supplement 1 show databases and codes to define variables. ICES is an independent, nonprofit research institute whose legal status under Ontario’s health information privacy law allows it to collect and analyze health care and demographic data, without informed consent, for health system evaluation and improvement. The datasets were linked using unique encoded identifiers and analyzed at ICES Western. In addition to outcomes described below, other variables included sociodemographic variables (age at diagnosis, sex, rural status, and income quintile), treatment era (1993-2001, 2002-2010, and 2011-2020), and for CCS, cancer characteristics (including cancer type, treatment duration, nephrectomy, radiation, stem cell transplantation, and some nephrotoxic chemotherapies).^56,57^ We looked back 1 year (or to birth if aged ≤12 months) before treatment start (for CCS; equivalent for comparator cohorts) for preexisting comorbidities (cardiac, chronic liver disease, or diabetes) and the Pediatric Medical Complexity Algorithm classification and 1 to 13 months before treatment start date for hospitalizations, emergency department, primary care, and specialist visits (eTable 2 in Supplement 1).^58^

### Outcomes

The primary composite outcome was incident CKD or hypertension based solely on diagnosis and procedure codes, as previously described.^51,52,53^ Laboratory data to ascertain or stage CKD by international guidelines^59^ were unavailable. Secondary outcomes included incident CKD, incident hypertension, incident CKD and hypertension, and incident end-stage kidney disease (ESKD; chronic dialysis or kidney transplant). To avoid underestimating outcomes, we included outcomes occurring more than 90 days after cancer treatment start date (CKD requires 3 months to be diagnosed)^59^; for patients whose outcome occurred before treatment end (index) date, the outcome was assigned as being present at index start. Diagnosis and procedures codes for defining CKD, ESKD, and hypertension are shown in eTable 2 in Supplement 1.

### Statistical Analysis

Standardized differences (≥10% is considered significant) were used to compare baseline differences between CCS and comparator cohorts.^60^ Numbers of CCS matched to each comparator cohort differed slightly; thus, all results and number of individuals of CCS vs comparator cohorts were reported separately. We calculated follow-up time and event rate per 1000 person-years of all outcomes and cumulative incidence of outcomes at 1 year, 3 years, 5 years, 10 years, and maximum follow-up (predefined) and created cumulative incidence function curves. We performed Fine and Gray subdistribution hazard modeling (reporting hazard ratios [HRs] with 95% CIs, accounting for competing risks of death and new cancer diagnosis or relapse) to evaluate associations of cancer treatment with outcomes. We repeated analyses adjusting for cardiac disease, liver disease, and diabetes. For the primary outcome, we performed cause-specific hazard modeling for comparison. When the proportionality assumption was violated, the hazard model was stratified by follow-up time using data-driven time periods (follow-up <1 year or ≥1 year). For the primary outcome, we performed stratified adjusted analyses by sex, age groups (≤1 year, >1 year to ≤12 years, and >12 years), cancer type (4 categories), stem cell transplant, cisplatin, ifosfamide and high-dose methotrexate use, nephrectomy, radiation use, and era (defined previously), reporting the interaction term *P* value. *P* < .05 was considered statistically significant. We conducted analyses with SAS version 9.4 (SAS Institute) at the ICES Western facility (London, Ontario, Canada) from March 2021 to August 2024.

## Results

### Study Population Baseline and Cancer Characteristics

After exclusions (Figure 1), there were 10 182 CCS (median [IQR] age at diagnosis, 7 [3-13] years; 5529 male [54.3%]; median [IQR] follow-up time, 8 [2-15] years) matched to 40 728 hospitalization cohort patients (median [IQR] age at diagnosis, 7 [2-12] years; 5529 male [weighted percentage, 54.3%]; median [IQR] follow-up time, 11 [6-18] years) and 8849 CCS (median [IQR] age at diagnosis, 5 [2-11] years; 4825 male [54.5%]; median [IQR] follow-up time, 7 [2-14] years) matched to 35 307 GP cohort individuals (median [IQR] age at diagnosis, 6 [2-11] years; 4825 male [weighted percentage, 54.5%]; median [IQR] follow-up time, 10 [5-16] years) (Table 1 ). Matching characteristics were similar between cohorts (Table 1 and eTable 3 in Supplement 1). However, the CCS cohort had more comorbidities and health care utilization before cohort entry than both comparator cohorts. The 3 most common cancers in the CCS cohort were leukemia (2948 patients [29.0%]), central nervous system neoplasms (2123 patients [20.9%]), and lymphoma (1583 patients [15.5%]). Median (IQR) treatment duration was 176 (90-712) days, and 4978 patients (48.9%) received well-known nephrotoxic chemotherapies (eTable 4 in Supplement 1).

**Table 1.** Cohort Characteristics of CCS and 2 Matched Comparator Cohorts

Characteristic	CCS cohort participants, No. (%) (n = 10 182)	Hospitalization cohort participants, No. (weighted %) (n = 40 728)	Standardized difference, %^a^	CCS cohort participants, No. (%) (n = 8849)	GP cohort participants, No. (weighted %) (n = 35 307)	Standardized difference, %^a^
						
Demographics						
Age at diagnosis						
Median (IQR), y	7 (3-13)	7 (2-12)	0	5 (2-11)	6 (2-11)	2
≤1 y	1649 (16.2)	2060 (20.2)	10^b^	1649 (18.6)	1664 (18.8)	1
>1 y and ≤12 y	5514 (54.2)	5090 (50.0)	8	5092 (57.5)	5141 (58.1)	1
>12 y	3019 (29.7)	3032 (29.8)	0	2108 (23.8)	2043 (23.1)	2
Age at index date						
Median (IQR), y	8 (4-13)	8 (4-13)	0	7 (4-12)	7 (4-12)	2
≤1 y	1048 (10.3)	1050 (10.3)	0	1048 (11.8)	1063 (12.0)	1
>1 y and ≤12 y	5620 (55.2)	5608 (55.1)	0	5299 (59.9)	5329 (60.2)	1
>12 y	3514 (34.5)	3525 (34.6)	0	2502 (28.3)	2457 (27.8)	1
Sex						
Male	5529 (54.3)	5529 (54.3)	0	4825 (54.5)	4825 (54.5)	0
Female	4653 (54.7)	4653 (45.7)	0	4024 (45.5)	4024 (45.5)	0
Rural status^c^	1103 (10.8)	1103 (10.8)	0	924 (10.4)	924 (10.4)	0
Income quintile^d^						
1	1833 (18.0)	1833 (18.0)	0	1636 (18.5)	1636 (18.5)	0
2	1869 (18.4)	1869 (18.4)	0	1618 (18.3)	1618 (18.3)	0
3	2084 (20.5)	2084 (20.5)	0	1815 (20.5)	1815 (20.5)	0
4	2231 (21.9)	2231 (21.9)	0	1924 (21.7)	1924 (21.7)	0
5	2165 (21.3)	2165 (21.3)	0	1856 (21.0)	1856 (21.0)	0
ICU admission between treatment start (or equivalent in comparator cohorts) and index date	248 (2.4)	881 (2.2)	0	229 (2.6)	324 (0.9)	13^b^
Era						
1993-2001	2655 (26.1)	2646 (26.0)	0	1590 (18.0)	1464 (16.5)	4
2002-2010	3495 (34.3)	3498 (34.4)	0	3229 (36.5)	3336 (37.7)	2
2011-2020	4032 (39.6)	4038 (39.7)	0	4030 (45.5)	4049 (45.8)	1
Comorbidities^e^						
Cardiac disease	652 (6.4)	441 (4.3)	0.09	610 (6.9)	53 (0.6)	0.34^b^
Chronic liver disease	261 (2.6)	65 (0.6)	0.16^b^	241 (2.7)	13 (0.1)	0.22^b^
Diabetes	124 (1.2)	227 (2.2)	0.08	114 (1.3)	12 (0.1)	0.14^b^
PMCA^f^						
Nonchronic	7178 (70.5)	9608 (94.4)	0.66^b^	6282 (71.0)	8782	0.86^b^
Noncomplex chronic	452 (4.4)	403 (4.0)	0.02	401 (4.5)	50	0.25^b^
Complex chronic	2552 (25.1)	171 (1.7)	0.73^b^	2166 (24.5)	17	0.79^b^
Baseline health care utilization^g^						
Hospitalizations						
0	8488 (83.4)	8936 (87.8)	13^b^	7311 (82.6)	8255 (93.3)	33^b^
≥1	1694 (16.6)	1246 (12.2)	13^b^	1538 (17.4)	594 (6.7)	33^b^
Emergency department visits						
0	7263 (71.3)	7516 (73.8)	6	6032 (68.2)	7656 (86.5)	45^b^
1	1642 (16.1)	1545 (15.2)	2	1583 (17.9)	829 (9.4)	25^b^
≥2	1277 (12.5)	1122 (11.0)	5	1234 (13.9)	364 (4.1)	35^b^
Primary care visits						
0	1299 (12.8)	1538 (15.1)	7	1104 (12.5)	3642 (41.2)	68^b^
1	1043 (10.2)	1273 (12.5)	7	877 (9.9)	1129 (12.8)	9
≥2	7840 (77.0)	7371 (72.4)	11^b^	6868 (77.6)	4079 (46.1)	69^b^
Specialist visits						
0	7595 (74.6)	8385 (82.4)	19^b^	6668 (75.4)	8341 (94.3)	55^b^
1	1080 (10.6)	930 (9.1)	5	931 (10.5)	333 (3.8)	26^b^
≥2	1507 (14.8)	868 (8.5)	20^b^	1250 (14.1)	175 (2.0)	46^b^

Abbreviations: CCS, childhood cancer survivors; ICU, intensive care unit; GP, general population; PMCA, pediatric medical complexity algorithm.

^a^ Standardized differences were used to compare CCS with the hospitalization and GP cohorts (referent groups). A standardized difference greater than 10% is considered a meaningful difference.

^b^ Statistically significant (standardized difference >10%).

^c^ Rural status was defined as residence within a community less than 10 000 persons.

^d^ Income quintile was defined as neighborhood income quintile by postal code.

^e^ Comorbidities diagnosed 1 year before cancer treatment start dates (or equivalent dates in the comparator cohorts) were considered.

^f^ PMCA classification is a validated algorithm used to classify children with chronic disease according to medical complexity using administrative data from up to 3 years before treatment start date (or equivalent in comparator cohorts).

^g^ Health care utilization was evaluated 1 month to 13 months prior to cancer treatment start date in the CCS cohort, to hospitalization discharge date in the hospitalization cohort, and to a randomly assigned date (based on distribution of dates for CCS cohort) in the GP cohort.

### Cumulative Incidence of Kidney Outcomes

The cumulative incidence in the CCS cohort of CKD or hypertension was 4.80% (95% CI, 4.40%-5.23%) by 1 year and 9.99% (95% CI, 9.37%-10.63%) by 10 years after cancer therapy end (Table 2); the overall cumulative incidence of CKD among CCS was 8.07% (95% CI, 7.07%-9.15%). Median (IQR) age at CKD or hypertension diagnosis for CCS vs hospitalization cohorts was 15 (7-20) vs 20 (15-26) years, respectively, and 12 (6-17) vs 18 (13-22) years for CCS vs GP cohorts, respectively. During the 27-year observation period, cumulative incidence of the primary outcome was 20.85% (95% CI, 18.75%-23.02%) vs 16.47% (95% CI, 15.21%-17.77%) for the CCS vs hospitalization cohorts and 19.24% (95% CI, 15.99%-22.73%) vs 8.05% (95% CI, 6.76%-9.49%) for the CCS vs GP cohorts (Table 2). In the CCS cohort, only 1.73% of CKD or hypertension diagnoses first appeared during cancer therapy. Cumulative incidence of hypertension diagnosis was 15.86% (95% CI, 13.88%-17.96%) vs 15.13% (95% CI, 13.90%-16.42%) in the CCS vs hospitalization cohorts and 12.98% (95% CI, 10.08-16.25) vs 7.83% (95% CI, 6.53%-9.27%) in the CCS vs GP cohorts. Cumulative incidence function curves (eFigure in Supplement 1) visually demonstrate that the majority of outcomes in the CCS group occurred within 2 years after treatment end.

**Table 2.** Event Rate and Cumulative Incidence of Outcomes in CCS and 2 Comparator Cohorts

Cohort	Total follow-up time, y	Event rate per 1000 person-years	Cumulative incidence by No. of years since cancer therapy, % (95% CI)				
			1	3	5	10	Maximum (27 y)
**Primary outcome**							
CKD or HTN							
Hospitalization vs CCS							
Hospitalization	489 621	5.17	0.76 (0.68-0.85)	1.44 (1.33-1.56)	2.13 (1.99-2.28)	4.08 (3.86-4.29)	16.47 (15.21-17.77)
CCS	93 058	12.71	4.80 (4.40-5.23)	6.32 (5.85-6.80)	7.44 (6.93-7.97)	9.99 (9.37-10.63)	20.85 (18.75-23.02)
GP vs CCS							
GP	383 612	2.07	0.20 (0.16-0.26)	0.47 (0.41-0.55)	0.72 (0.63-0.82)	1.65 (1.50-1.81)	8.05 (6.76-9.49)
CCS	75 007	12.20	4.84 (4.40-5.30)	6.35 (5.86-6.88)	7.36 (6.82-7.93)	9.59 (8.94-10.27)	19.24 (15.99-22.73)
**Secondary outcomes**							
CKD and HTN							
Hospitalization vs CCS							
Hospitalization	491 141	0.40	0.06 (0.04-0.08)	0.10 (0.08-0.14)	0.17 (0.13-0.22)	0.31 (0.26-0.38)	1.28 (1.00-1.62)
CCS	94 579	1.52	0.42 (0.31-0.57)	0.59 (0.46-0.76)	0.74 (0.59-0.93)	1.14 (0.93-1.38)	3.22 (2.46-4.13)
GP vs CCS							
GP	383 885	0.07	0.01 (0.00-0.02)	0.02 (0.01-0.04)	0.02 (0.01-0.05)	0.06 (0.04-0.11)	0.23 (0.12-0.42)
CCS	76 247	1.35	0.43 (0.31-0.59)	0.59 (0.44-0.77)	0.73 (0.56-0.93)	1.12 (0.89-1.38)	2.21 (1.63-2.94)
CKD							
Hospitalization vs CCS							
Hospitalization	505 766	0.99	0.24 (0.20-0.29)	0.40 (0.34-0.46)	0.57 (0.50-0.65)	0.95 (0.85-1.06)	2.55 (2.21-2.93)
CCS	97 726	5.67	3.05 (2.73-3.40)	3.81 (3.45-4.20)	4.20 (3.82-4.61)	4.99 (4.56-5.44)	8.07 (7.07-9.15)
GP vs CCS							
GP	387 750	0.19	0.02 (0.01-0.04)	0.06 (0.04-0.09)	0.09 (0.06-0.13)	0.19 (0.15-0.25)	0.47 (0.32-0.65)
CCS	78 124	5.98	3.04 (2.70-3.41)	3.84 (3.46-4.26)	4.21 (3.80-4.65)	5.00 (4.54-5.50)	8.47 (6.95-10.17)
HTN							
Hospitalization vs CCS							
Hospitalization	491 969	4.53	0.58 (0.51-0.66)	1.15 (1.05-1.26)	1.73 (1.60-1.87)	3.44 (3.24-3.64)	15.13 (13.90-16.42)
CCS	94 983	8.24	2.24 (1.97-2.54)	3.19 (2.86-3.55)	4.06 (3.68-4.47)	6.23 (5.73-6.76)	15.86 (13.88-17.96)
GP vs CCS							
GP	383 911	1.95	0.19 (0.15-0.24)	0.44 (0.37-0.51)	0.66 (0.58-0.75)	1.52 (1.38-1.67)	7.83 (6.53-9.27)
CCS	76 690	7.32	2.31 (2.01-2.63)	3.21 (2.86-3.60)	3.97 (3.57-4.40)	5.82 (5.29-6.37)	12.98 (10.08-16.25)
ESKD							
Hospitalization vs CCS							
Hospitalization	509 378	0.09	0.00 (0.00-0.01)	0.01 (0.00-0.03)	0.01 (0.01-0.03)	0.05 (0.03-0.09)	0.35 (0.23-0.53)
CCS	100 345	0.24	0.10 (0.05-0.18)	0.13 (0.07-0.22)	0.15 (0.09-0.25)	0.18 (0.11-0.29)	0.53 (0.28-0.95)
GP vs CCS							
GP	388 169	0.00-0.01^a^	0.00 (0.00-0.00)	0.00 (0.00-0.00)	0.00 (0.00-0.00)	0.01 (0.00-0.03)	0.08 (0.02-0.29)
CCS	80 281	0.22	0.10 (0.05-0.19)	0.13 (0.07-0.22)	0.15 (0.09-0.26)	0.19 (0.11-0.31)	0.29 (0.17-0.49)

Abbreviations: CCS, childhood cancer survivors; CKD, chronic kidney disease; ESKD, end-stage kidney disease; GP, general population; HTN, hypertension.

^a^ In order to prevent reidentification of individual data, a range is shown for event rate (being no more than 0.01 events per 1000 person years), instead of a unique value, because the number of events was less than 6, as per local privacy protection rules.

### Association of CCS Status With Kidney Outcomes

Compared with the hospitalization and GP cohorts, the CCS cohort had an approximate overall 2-fold (adjusted HR [aHR], 2.00; 95% CI, 1.86-2.14) and 4-fold (aHR, 4.71, 95% CI, 4.27-5.19) higher adjusted risk of CKD or hypertension, respectively (Table 3). The magnitude of these associations were largest when follow-up time was less than 1 year (aHR, 6.45; 95% CI, 5.78-7.19 for CCS vs hospitalization cohort; aHR, 23.57; 95% CI, 19.57-28.39 for CCS vs GP cohort) (Table 3); however, the proportionality assumption was not always met for the less than 1 year time period. The CCS cohort had increased risk for all secondary outcomes, except ESKD for follow-up time of 1 or more years (Table 3).

**Table 3.** Outcomes in CCS vs 2 Comparator Cohorts for All Years and Stratified by Less Than 1 vs 1 or More Years of Follow-Up Time^a^

Cohort	Participants, No.	Events, No.	All patients				Follow-up time <1 y, aHR (95% CI)^b^	*P* value	Follow-up time ≥1 y, aHR (95% CI)^b^	*P* value
			HR (95% CI)	*P* value	aHR (95% CI)^b^	*P* value				
**Primary outcome**										
CKD or HTN										
Hospitalization vs CCS										
Hospitalization	40 728	2530	1 [Reference]	NA	1 [Reference]	NA	1 [Reference]	NA	1 [Reference]	NA
CCS	10 182	1183	2.00 (1.86-2.14)	<.001	2.00 (1.86-2.14)^c^	<.001	6.45 (5.78-7.19)	<.001	1.35 (1.22-1.50)	<.001
GP vs CCS										
GP	35 307	793	1 [Reference])	NA	1 [Reference]	NA	1 [Reference]	NA	1 [Reference]	NA
CCS	8849	915	4.82 (4.38-5.31)	<.001	4.71 (4.27-5.19)^c^	<.001	23.57 (19.57-28.39)	<.001	2.79 (2.46-3.15)	<.001
**Secondary outcomes**										
CKD and HTN										
Hospitalization vs CCS										
Hospitalization	40 728	197	1 [Reference]	NA	1 [Reference]	NA	1 [Reference]	NA	1 [Reference]	NA
CCS	10 182	144	3.04 (2.45-3.77)	<.001	3.29 (2.64-4.09)	<.001	8.11 (5.53-11.89)	<.001	2.63 (2.01-3.44)	<.001
GP vs CCS										
GP	35 307	27	1 [Reference]	NA	1 [Reference]	NA	1 [Reference]	NA	1 [Reference]	NA
CCS	8849	103	15.50 (10.18-23.61)	<.001	15.26 (9.96-23.37)	<.001	49.90 (38.64-64.44)	<.001	10.90 (6.72-17.69)	<.001
CKD										
Hospitalization vs CCS										
Hospitalization	40 728	500	1 [Reference]	NA	1 [Reference]	NA	1 [Reference]	NA	1 [Reference]	NA
CCS	10 182	554	4.61 (4.08-5.19)	<.001	4.73 (4.19-5.35)	<.001	13.32 (11.21-15.81)	<.001	2.62 (2.15-3.19)	<.001
GP vs CCS										
GP	35 307	73	1 [Reference]	NA	1 [Reference]	NA	1 [Reference]	NA	1 [Reference]	NA
CCS	8849	467	26.02 (20.33-33.29)	<.001	25.09 (19.56-32.19)	<.001	130.86 (84.77-202.00)	<.001	12.04 (8.96-16.19)	<.001
HTN										
Hospitalization vs CCS										
Hospitalization	40 728	2230	1 [Reference]	NA	1 [Reference]	NA	1 [Reference]	NA	1 [Reference]	NA
CCS	10 182	783	1.46 (1.35-1.59)	<.001	1.46 (1.35-1.59)	<.001	3.91 (3.41-4.49)	<.001	1.17 (1.05-1.30)	.005
GP vs CCS										
GP	35 307	747	1 [Reference]	NA	1 [Reference]	NA	1 [Reference]	NA	1 [Reference]	NA
CCS	8849	561	3.05 (2.73-3.40)	<.001	3.00 (2.68-3.36)	<.001	12.06 (9.84-14.78)	<.001	2.10 (1.83-2.41)	<.001
ESKD										
Hospitalization vs CCS										
Hospitalization	40 728	45	1 [Reference]	NA	1 [Reference]	NA	1 [Reference]	NA	1 [Reference]	NA
CCS	10 182	24	2.19 (1.33-3.59)	.002	2.41 (1.44-4.03)	.001	43.74 (5.66-337.77)	<.001	1.45 (0.74-2.85)	.28
GP vs CCS										
GP	35 307	<6	1 [Reference]	NA	1 [Reference]	NA	NA	NA	NA	NA
CCS	8849	18	14.16 (5.22-38.42)	<.001	14.13 (5.17-38.61)	<.001	NA	NA	NA	NA

Abbreviations: aHR, adjusted hazard ratio; CCS, childhood cancer survivors; CKD, chronic kidney disease; ESKD, end-stage kidney disease; GP, general population; HR, hazard ratio; HTN, hypertension; NA, not applicable.

^a^ Analyses accounted for the competing risks of death and new cancer diagnosis or relapse. The proportionality assumption was violated, so proportional hazard modeling was stratified by follow-up time using data-driven time periods (follow-up < or ≥1 year). For the greater than 1 year time period, proportionality assumption was not always met.

^b^ Adjusted for cardiac disease, liver disease, and diabetes.

^c^ Cause-specific hazard modeling showed that cause-specific HR for the primary outcome was 5.12 (95% CI, 4.87-5.39) for the CCS vs hospitalization cohort and 12.76 (95% CI, 11.83-13.77) for the CCS cohort vs general population cohort.

### Stratified Analysis

eTable 5 in Supplement 1 shows that compared with the GP cohort, the adjusted risk for CKD or hypertension was higher in patients with several nephrotoxic therapies (interaction terms were statistically significant for stem cell transplant, cisplatin, nephrectomy and radiation, while there was no difference in risk between patients who did vs did not receive ifosfamide or high-dose methotrexate) and in patients treated in the latest era (2010-2020) vs earlier eras, consistent with CCS event rates occurring early after cancer therapy end. There was no difference in adjusted risk of outcomes across sex or age groups (eTable 5 in Supplement 1). Stratified analyses were similar in the CCS vs hospitalization cohorts (eTable 5 in Supplement 1) except that males (vs females) and younger (vs >12 years) patients were at higher risk for CKD or hypertension, as were CCS receiving high-dose methotrexate (vs not) (eTable 5 in Supplement 1).

## Discussion

In this cohort study, we found that CCS had increased risk for CKD or hypertension over a maximum of 27 years follow-up, relative to hospitalized children and the Ontario GP of children; outcomes became evident within the first year after cancer treatment. These results strengthen the hypothesis that CCS require monitoring for blood pressure and kidney health soon after cancer treatment is complete and ongoing into adulthood.

Data on long-term CKD and hypertension in CCS have been limited by small sample sizes and requiring prolonged follow-up. The overall cumulative incidence of CKD in our study was 8% among CCS, but the true incidence may be higher, because we used administrative data to define CKD. In adults, administrative data-defined CKD and hypertension diagnoses have been shown to be highly specific (ie, if diagnosis is ascertained, it is likely to be present) but not sensitive; recent pediatric studies showed similar characteristics.^41,42,43,44,45,46,47^ Previous studies reported that CCS had an ESKD incidence of 0.5% to 1.7% by 30-year follow-up, which aligns with the ESKD cumulative incidence in this study.^7,34,38^ Mild to moderate CKD is associated with many complications and may progress to ESKD.^13,15,16,17^ Other studies, with smaller sample sizes and mostly without comparator groups, have evaluated non–end-stage CKD, including low glomerular filtration rate (GFR) and/or albuminuria.^8,10,12,61,62^ We found that cumulative incidence of outcomes increased over time, but began in the first few years after cancer therapy (Table 2 and eFigure in Supplement 1), which has major implications. To detect and mitigate complications of CKD and hypertension, screening must begin soon after cancer therapy. CCS may be exposed to many acute kidney injury (AKI) episodes; great endeavors to prevent or treat AKI have been studied, with a goal to prevent long-term kidney damage.^63,64,65,66^ CCS should figure prominently in current international endeavors aiming to improve follow-up of children (not specifically with cancer) who develop AKI,^67,68,69^ by pursuing collaboration with international pediatric cancer stakeholder groups.

Hypertension is a major risk factor for CKD, CVD, and mortality.^19,20,21,25^ Previous studies reporting hypertension in CCS were smaller and varied in design, although incidence was similar to our finding of approximately 15%.^9,37,61^ Hypertension was also more common than CKD was in comparator cohorts. One previous study^37^ showed similar prevalence of hypertension in CCS and controls. Hypertension is, thus, very common in CCS, and likely associated with several factors including obesity, another known late morbidity of CCS.^27,70^ Our findings support that CCS must be considered as a high CVD risk group requiring primary and secondary prevention.

Early treatment of CKD and hypertension can mitigate disease progression and decrease CVD risk.^22,23,24,25,30,31,32^ CCS have increased CVD risk.^4,26,27,28^ Several CCS late effects surveillance guidelines do not include kidney outcomes (including albuminuria assessment, an important CKD component).^39,40,71^ Moreover, existing CCS CKD and hypertension outcomes surveillance recommendations are nonspecific, with poor agreement on which patients to screen, with what tests, and how frequently and for how long to test. Global initiatives (eg, International Guidelines Harmonization Group for Late Effects of Childhood Cancer) may overcome these problems.^72^ However, efforts should be made to harmonize existing pediatric hypertension and CKD guidelines with CCS surveillance guidelines.^19,59^

Throughout cancer therapy, kidney damage occurs via many mechanisms (eg, different types of AKI, kidney tubular cell or microvascular damage, or other mechanisms), which are challenging to evaluate using administrative health care data; however, stem cell transplant, radiation, and certain chemotherapies (eg, cisplatin) are known to cause kidney damage via several mechanisms.^8,73,74,75^ Our large sample size allowed performance of stratified analyses by these variables; CCS with these exposures (vs without) were in general at even higher risk for the primary outcome, suggesting they may help risk-stratify CCS for CKD or hypertension. There were several exposures we could not evaluate (eg, anthracyclines, which cause cardiac effects and secondary hypertension,^76^ and thrombotic microangiopathy) as contributors to our study outcomes. However, research should focus on understanding injury mechanisms, with a goal to propose cause-specific treatment approaches for post–cancer therapy CKD and hypertension.

### Strengths and Limitations

To our knowledge, this is the largest cohort evaluation of CCS kidney outcomes after cancer therapy.^6,28^ Thirty percent of the Ontario population are immigrants from many countries, providing a diverse ethnic background.^48^ Including 2 large, matched comparator cohorts strengthened our conclusion that CCS are at much higher risk than other children for kidney outcomes. The use of administrative data from linked databases minimized cohort misclassification, loss to follow-up, and false-positive outcomes.^41,43,47^

However, this study also has limitations. Diagnosing pediatric CKD and hypertension using administrative data (and not laboratory data or blood pressure) is very specific but not sensitive. Thus, outcomes incidences reported likely underestimate true incidences^41,42^; this is important to highlight if using these data to justify resource allocation or future research. We were unable to differentiate CKD manifestations (eg, low GFR or albuminuria) and stages, which limits the ability to use our data to specifically comment on severity of CKD, likelihood of progression, and specific monitoring (ie, GFR vs proteinuria) that would be most appropriate for CCS. We surmise that had we been able to use laboratory data to define CKD severity, this information may have been biased (indication bias) because current guidelines provide poor guidance on kidney health and blood pressure follow-up. Although we performed matched analyses and further adjusted for several comorbidities, we were not able to confidently adjust for other possible risk factors such as obesity, family history, smoking, or key sociodemographic factors (eg, race and ethnicity), and we did not consider health events occurring between treatment end and outcome ascertainment. Each of these factors may be highly relevant in the context of long-term CVD risk assessment and may be better suited for prospective evaluative research. This study primarily evaluated burden and risk rather than risk factors for kidney health issues; such analyses will need to consider validity of defining proposed risk factors in administrative health care data and such data may not be as easily available (eg, other nephrotoxic drugs, contrast, and intensive care unit admission). Future analyses will hopefully further elucidate modifiable risk factors and predictors for late kidney health issues. We were also not able to include AKI during cancer therapy as a variable in the current analysis comparing risks with comparator cohorts; this will prove challenging because AKI can occur multiple times over a prolonged period of time for CCS, as opposed to a single AKI episode occurring during a hospitalization in most hospitalized children. Future research should elucidate how best to evaluate the association of AKI with long-term kidney health in CCS relative to other at-risk children.

## Conclusions

Future research should attempt to refine which CCS are at highest risk for kidney health outcomes in order to develop a cost-effective approach to screening for CKD and hypertension. Additional studies examining high-risk CCS subgroups, and prospective studies using laboratory-defined outcomes, may overcome some of our study limitations and complete the picture of late kidney outcomes in CCS. Increasing specificity and action-oriented guidance of CCS surveillance guidelines, by collaboration of kidney and cancer stakeholder organizations, may help mitigate CKD and hypertension-associated morbidity and mortality.

## Notes

Supplement 1. **eTable 1.** Databases Used for the Study **eTable 2.** Administrative Codes Used for Exclusions, Follow-Up Termination, Baseline Characteristics and Outcomes **eTable 3.** Cohort Characteristics of CCS and Two Comparator Cohorts (Before Matching) **eFigure.** Cumulative Incidence Function Curves for CKD, Hypertension and CKD or Hypertension (Primary Outcome) in CCS Versus Comparator Cohorts **eTable 4.** Cancer Characteristics of CCS **eTable 5.** Analyses by Demographic and Clinical Subgroups: Adjusted Hazard Ratio for CKD or HTN in CCS Compared With Two Comparator Cohorts

Supplement 2.
Data Sharing Statement
