# Supplementary material for: Chronic Kidney Disease or Hypertension After Childhood Cancer
Source: JAMA Netw Open. 2025 May 19;8(5):e258199. doi: 10.1001/jamanetworkopen.2025.8199 (PMC12090035; doi:10.1001/jamanetworkopen.2025.8199)
Supplement: Supplement 1. — eTable 1. Databases Used for the Study eTable 2. Administrative Codes Used for Exclusions, Follow-Up Termination, Baseline Characteristics and Outcomes eTable 3. Cohort Characteristics of CCS and Two Comparator Cohorts (Before Matching) eFigure. Cumulative Incidence Function Curves for CKD, Hypertension and CKD or Hypertension (Primary Outcome) in CCS Versus Comparator Cohorts eTable 4. Cancer Characteristics of CCS eTable 5. Analyses by Demographic and Clinical Subgroups: Adjusted Hazard Ratio for CKD or HTN in CCS Compared With Two Comparator Cohorts [file jamanetwopen-e258199-s001.pdf]

Supplemental Online Content

Lebel A, Chanchlani R, Cockovski V, et al. Chronic kidney disease or hypertension after childhood cancer. *JAMA Netw. Open*. 2025;8(5):e258199.
doi:10.1001/jamanetworkopen.2025.8199

eTable 1. Databases Used for the Study

eTable 2. Administrative Codes Used for Exclusions, Follow-Up Termination, Baseline Characteristics and Outcomes

eTable 3. Cohort Characteristics of CCS and Two Comparator Cohorts (Before Matching)

eFigure. Cumulative Incidence Function Curves for CKD, Hypertension and CKD or Hypertension (Primary Outcome) in CCS Versus Comparator Cohorts

eTable 4. Cancer Characteristics of CCS

eTable 5. Analyses by Demographic and Clinical Subgroups: Adjusted Hazard Ratio for CKD or HTN in CCS Compared With Two Comparator Cohorts

This supplemental material has been provided by the authors to give readers additional information about their work.

© 2025 Lebel A et al. *JAMA Network Open*

eTable 1: Databases used for the study

Database		Data	Use
	Ontario Census Area Profiles	Demographic data	Demographic characteristics
**CIHI-DAD**	Canadian Institute of Health Information Discharge Abstract Database	Hospitalization data	Cohort build, exclusions, baseline characteristics, outcomes
**CIHI-SDS**	Canadian Institute of Health Information Same Day Surgery Database	Hospitalization data	Cohort build, exclusions, baseline characteristics
**CORR**	Canadian Organ Replacement Registry	Chronic dialysis and transplantation data	Exclusions, outcomes
**IPDB**	ICES Physician database	Physician data	Baseline characteristics
**MOMBABY**	Linked Delivering Mother and Newborns	Birth hospitalization data	Exclusions
**CIHI-NACRS**	Canadian Institute of Health Information National Ambulatory Care Reporting System	Ambulatory care data	Cohort build, exclusions, baseline characteristics, outcomes
**OCR**	Ontario Cancer Registry	Adult cancer diagnoses	Exclusions, follow-up termination
**OHIP**	Ontario Health Insurance Plan Claims Database	Diagnoses, billing codes	Cohort build, exclusions, baseline characteristics, outcomes
**POGONIS**	Pediatric Oncology Group of Ontario Networked Information System	Pediatric cancer data	Cohort build, follow-up termination, cancer characteristics
**PCCF**	Postal Code Conversion File	Demographic data	Demographic characteristics
**RPDB**	Registered Persons Database: *RPDB is a database of all current and former residents of Ontario that have a health card. It comes directly from the MOH and is very reliable for all permanent residents of Ontario, regardless of age.*	Demographic data	Demographic characteristics

© 2025 Lebel A et al. *JAMA Network Open*

eTable 2: Administrative codes used for exclusions, follow-up termination, baseline characteristics and outcomes

Database	Variable Name	Code or Algorithm
Childhood Cancer Cohort Inclusion		
POGONIS.PATIENT	PATIENTID	
	IKN	
	VALIKN	
POGONIS.DIAGNOSIS	DIAG_DATE	
	EVENT_ID	
	PATIENT_ID	
At Risk-Cohort Inclusion		
CIHI-DAD	ADMDATE	
	DDATE	
Healthy Cohort Inclusion		
RPDB	BDATE	
Exclusion Criteria for CCS Cohort		
No Treatment Initiation		
POGONIS.TREATMENT_PLAN	START_DATE	
	PATIENT_ID	
	EVENT_ID	
Did not complete treatment during study period		
POGONIS.TREATMENT_PLAN	END_DATE	
TOPOST.CHEMOTHERAPY	CHEMO_YEAR	
TOPOST.EVENT	EVENT_DATE	
	EVENT_NAME	
TOPOST.BMT	BMT_DATE	
Exclusion Criteria for all three cohorts		
Invalid or missing date of birth or sex or non-Ontario resident		
RPDB	SEX	
	BDATE	
	PRCDDABLK	35

© 2025 Lebel A et al. *JAMA Network Open*

0 years < Age > 18 years		
RPDB	AGE	
Death on or before index date		
RPDB	DTHDATE	DTHDATE<=index date
Previous cancer or multiple same day diagnosis		
POGONIS.PATIENT	IKN	CCS Cohort: DIAGDATE < index diagnosis date<br>At Risk/Healthy Cohort: DIAGDATE < index date
	PATIENT_ID	
POGONIS.DIAGNOSIS	PATIENT_ID	
	DIAGDATE	
Previous non-renal organ transplant		
CORR.RECIPIENT_TREATMENT	TREATMENT_CODE	171
	TRANSPLANTED_ORGAN_TYPE_CODE[1-3]	20, 21, 22, 23, 29, 40, 41, 42, 48, 49, 30, 43, 90, 99, 50, 51, 52, 53, 54, 55
CIHI-DAD, NACRS	ICD9	V427, V426, V421, 9968, E878
	ICD10	T86400, T86401, T86402, Z944, Z942, Z941, T862, T863, Z943, Z9482, Z9483, T868, Y830
	CCP	624, 6241, 6249, 455, 456, 495, 6481, 6484
	CCI	1OA85, 1GT85, 1GR85, 1HZ85, 1OK85TNXXK, 1OK85XTXXK, 1OK85XUXXK, 1OK85XVXXK
OHIP	Fee code	S294, S295, E765, G254, M155, M156, H802, R870, R874, J205
Prior renal organ transplant		
CORR.RECIPIENT_TREATMENT	TREATMENT_CODE	171
	TRANSPLANTED_ORGAN_TYPE_CODE[1-3]	10, 11, 12, 18, 18, 19
CIHI-DAD, NACRS	ICD9	V420
	ICD10	T861
	CCP	6759, 6743, 6750
	CCI	1PC85
OHIP	Fee code	E762, E769, E771, G347, G348, G408, G409, G412, S434, S435, Z631
Prior Dialysis		
CORR.RECIPIENT_TREATMENT	TREATMENT_CODE	Not in 171, 181

© 2025 Lebel A et al. *JAMA Network Open*

CIHI-DAD, NACRS	CCP	5195, 6698
	CCI	1PZ21
OHIP	Fee code	R849, G323, G325, G326, G860, G862, G865, G863, G866, G330, G331, G333, G861, G082, G083, G085, G090, G091, G092, G093, G094, G095, G096, G294, G295, G864, H540, H740
Prior Chronic Kidney Disease (not including chronic dialysis or renal transplant)		
CIHI-DAD, NACRS	ICD9	585, 586, 7944, 2504, 4030, 4031,4039, 4040, 4041, 4049, 5888, 5889
	ICD10	N03, N11, E102, E112, E132, E142, I12, I13, N08, N18, N19, R944
OHIP	Diagnostic code	403, 585
History of Hypertension		
OHIP	Diagnostic code	401, 402, 403
Baseline Characteristics		
Income Quintile		
RPDB	INCQUINT	
Rural Status		
RPDB	RURAL	
Gestational Age		
MOMBABY	B_GESTWKS_DEL	
Birthweight		
MOMBABY	B_WEIGHT	
Local Health Integration Network		
RPDB	LHIN	
Cardiac Disease		
CIHI-DAD	ICD9	402, 404, 425, 428, 5184, 514, 410, 411, 412, 413, 414, 4292, 4296, 4297,430, 431, 3623, 4330, 4331, 4332, 4333, 4338, 4339, 434, 435, 436, 4402, 4408,4 409, 4439, 5571, 4275, 7999, 4261, 4262, 4263, 4264, 4265, 4266, 4267,4268, 4269,427, 42741, 42742, 4273, 745, 746, 7470, 7471, 7472, 7473, 7474
	ICD10	I11, I13, I420, I425, I426, I427, I428, I429, I43, I50, I255, J81, I20, I21, I22, I23, I24, I25, Z955, Z958, Z959, R931, T822, I60, I600, I601, I602,

© 2025 Lebel A et al. *JAMA Network Open*

		I603, I604, I605, I606, I607, I608, I609, I61, I610, I611, I612, I613, I614, I615, I616, I618, I619, I630, I631, I632, I633, I634, I635, I638, I639, I64, H341, G450, G451, G452, G453, G458, G459, H340, I700, I702, I708, I709, I731, I738, I739, K551, I460, I461, I469, I 44, I45, I47, I48, I4900, I4901, I491, I492, I493, I494, I498, I499, Q20, Q21, Q22, Q23, Q24, Q25, Q26
	CCP	4961, 4962, 4963, 4964, 4802, 4803, 4809, 481, 5011, 5012, 5014, 5016, 5018, 5021, 5022, 5024, 5028, 503, 5036, 5038, 5125, 5126, 5129, 1732, 481, 482, 483, 4801, 4802, 4803, 4804, 4805, 4809, 4811, 4812, 4813, 4814, 4815, 4816, 4817, 4819, 4892 4893 4894, 4895, 4896, 4897, 4898, 4995, 4996, 4997 0345 0346, 0347, 0348, 0349, 4971, 4972, 4973, 4974 4987 4988, 5164, 470, 471 472, 473, 474, 475 476 477 478 479, 482, 483, 489, 495, 4811, 4812, 4813, 4814, 4815, 4816, 4817, 4819, 5024, 5034
	CCI	1HP53, 1HP55, 1HZ53GRFR, 1HZ53LAFR, 1HZ53SYFR, 1IJ50, 1IJ57GQ, 1IJ76, 1JE57, 1JE87LA, 1JM76NC, 1JM76NCXXN, 1KA50, 1KA76, 1KA87LA, 1KE57, 1KE76, 1KG76MI, 1KG76MZXXA, 1KG76MZXXN, 1KG87, 1IA87LA, 1IB87LA,1IC87LA, 1ID87LA, 1HZ30JN, 1HZ30JY, 1IJ26, 1IJ27, 1IJ5, 1IJ54, 1IJ54GQAZ, 1IJ57, 1IJ57GQ, 1IJ76, 2HZ28GPPL, 2HZ71GP,3HZ30GP, 3IJ30GP, 1HB53, 1HD53GRJA, 1HD54GRJA,1HD55, 1HZ09, 1HZ37,1HZ55, 1HZ53HAFS, 1HZ53LAFS, 1HZ53SYFS,1HZ53GRFS, 1HZ53GRFR, 1HZ53GRNK, 1HZ53GRNL, 1HZ53GRNM, 1HZ53GRNN, 1HZ53LAFR, 1HZ53SYFR, 1HZ54LANJ, 1HZ55GPFS, 1HZ55LAFS, 1HZ55QAFS, 2HZ07FS, 2HZ07NK, 2HZ07NL, 2HZ07NM, 2HZ07NR, 2HZ24GPKJ, 2HZ24GPKL, 2HZ24GPKM, 2HZ24GPXJ, 1HJ76,1HJ82,1HN71, 1HN80, 1HN87, 1HP71, 1HP76, 1HP78, 1HP80, 1HP82, 1HP83, 1HP87,1 HR71, 1HR80, 1HS80, 1HT80, 1HT89, 1HU80, 1HV80, 1HW78, 1HW79, 1HZ34, 1HZ37, 1HZ80,1HZ85, 1IF83, 1IJ76, 1IN84, 1KA76, 1LC84, 1LD84, 1IJ50, 1IJ57GQ, 1IJ76
OHIP	Diagnostic code	428, 410, 412, 413, 436, 432, 435, 427, 745, 746, 747

© 2025 Lebel A et al. *JAMA Network Open*

	Fee code	R701, R702, Z429, E626, E649, E672, N220, R780, R783, R784, R785, R786, R787, R791, R792, R794, R797, R798, R799, R800, R802, R803, R804, R805, R809, R813, R814, R815, R816, R817, R855, R856, R860, R861, R867, R875, R933, R934, R936, R937, E645, E646, E651, E652, E654, E655, G262, G296, G297, G298, G299, G300, G301, G304, G305, G306, G509, R741, R742, R743, Z434, Z448, E628, G115, G176, G177, G178, G179, G249, G259, G261, G303, G317, G321, R709, R752, R753, R761, Z412, Z428, Z429, Z431, Z433, Z435, Z437, Z443, Z444, Z445, E646, E647, E649, E650, E651, E652, E656, E658, E660,E661, E670, E671, E682, R700, R701, R702, R703, R704, R706, R709, R710, R711, R712, R713, R714, R715, R716, R717, R718, R720, R721, R722, R723, R724, R725, R726, R727, R728, R729, R730, R733, R734, R735, R736,R737,R738, R741, R742, R743, R746, R747, R748, R749, R754, R755, R756, R757, R758, R759, R762, R763, R768, R769, R770, R771, R772, R773, R774, R781, R830, R841, R857, R863, R870, R874, R876, R920, R921, R922, R923,R924, R925, R926, R927, R928, R929,R930, Z465, Z466, Z743, Z759, Z780, R742, R743, Z734, G298
Chronic Liver Disease		
CIHI-DAD	ICD9	702, 703, 704, 705, 706, 707, 708, 2750, 2751, 4560, 4561, 4562, 571, 573, 5712, 5715, 5716, 5722, 5723, 5724, 5728, 7824, 7891, 7895
	ICD10	B18, B19, B180, B181, B942, E830, E831, I85, I982, I983, K70, K703, K713, K714, K715, K717, K721, K729, K73, K74, K743, K744, K745, K746, K753, K754, K758, K759, K767, K76, K77, R17, R18, R160, R162,
OHIP	Diagnostic code	571, 573, 070
	Fee code	Z551, Z554
Diabetes		
CIHI-DAD	ICD9	250
	ICD10	E10, E11, E13, E14, E102, E112, E132, E142
OHIP	Diagnostic code	248, 250
	Fee code	G500, G514, G520, K045, K046, K029, K030, Q040
PMCA		

© 2025 Lebel A et al. *JAMA Network Open*

CIHI-DAD	ICD9	
	ICD10	
Hospitalizations		
CIHI-DAD	DDATE	
Emergency Department Visits		
NACRS	REGDATE	
Primary Care Physician Visits		
IPDB	MAINSPECIALTY	GP/FP, PEDIATRICS
OHIP	SPEC	0, 26
Specialist Visits		
IPDB	MAINSPECIALTY	PEDIATRIC SURGERY, PEDIATRIC CARDIOLOGY, PEDIATRIC CLINICAL IMMUNOLOGY,<br>PEDIATRIC CRITICAL CARE, PEDIATRIC EMERGENCY MEDICINE, PEDIATRIC ENDOCRINOLOGY,<br>PEDIATRIC GASTROENTEROLOGY, PEDIATRIC HEMATOLOGY/ONCOLOGY, PEDIATRIC INFECTIOUS DISEASES, PEDIATRIC NEPHROLOGY, PEDIATRIC NEUROLOGY, PEDIATRIC RESPIROLOGY, PEDIATRIC RHEUMATOLOGY, NEONATAL-PERINATAL MEDICINE, GENERAL SURGERY, CARDIAC SURGERY, NEUROSURGERY, ORTHOPEDIC SURGERY, THORACIC SURGERY, VASCULAR SURGERY, CARDIOLOGY, CLINICAL IMMUNOLOGY, CRITICAL CARE MEDICINE, EMERGENCY MEDICINE, F.P./EMERGENCY MEDICINE, ENDOCRINOLOGY, GASTROENTEROLOGY, HEMATOLOGY, MEDICAL ONCOLOGY, GYNECOLOGIC ONCOLOGY, RADIATION ONCOLOGY, NFECTIOUS DISEASES, NEPHROLOGY, NEUROLOGY, RESPIROLOGY, RHEUMATOLOGY, INTERNAL MEDICINE
OHIP	SPEC	03, 04, 06, 09, 12, 13, 15, 16, 17, 18, 41, 44, 46, 47, 48, 60, 61, 62, 64
Cancer Characteristics		
Primary Cancer Diagnosis		
POGONIS.DIAGNOSIS	ICCC_SUBGROUP_Code	*Leukemias, myeloproliferative diseases, and myelodysplastic diseases*

© 2025 Lebel A et al. *JAMA Network Open*

		Ia., Ib., Ic., Id., Ie., Iz.<br><br>*Lymphomas and reticuloendothelial neoplasms*<br>IIa., IIb., IIc., IId., IIe., IIz.<br><br>*CNS and miscellaneous intracranial and intraspinal neoplasms*<br>IIIa., IIIb., IIIc., IIId., IIIe., IIIz., IIIf.<br><br>*Neuroblastoma and other peripheral nervous cell tumors*<br>IVa., IVb., IVz.<br><br>*Retinoblastoma*<br>V.<br><br>*Renal Tumors*<br>VIa., VIb., VIc., VIz.<br><br>*Hepatic tumors*<br>VIIa., VIIb., VIIc.<br><br>*Malignant Bone Tumors*<br>VIIIa., VIIIb., VIIIc., VIIId., VIIIe.<br><br>*Soft tissue and other extraosseous sarcomas*<br>IXa., IXb., IXc., IXd., IXe., IXz.<br><br>*Germ cell tumors, trophoblastic tumors, and neoplasms of gonads*<br>Xa.,Xb.,Xc.,Xd., Xz.<br><br>*Other malignant epithelial neoplasms and malignant melanomas*<br>XIa., XIb., XIc., XId., XIe., XIf., XIz.

© 2025 Lebel A et al. *JAMA Network Open*

		Other and unspecified malignant neoplasms<br>XIIa., XIIb., XIIz.
Renal Cancer		
POGONIS.DIAGNOSIS	ICCC_SUBGROUP_CODE	VIa., VIb., VIc., VIz.
Constitutional Chromosomal Abnormalities		
POGONIS.DIAGNOSIS	PRED_CONDITIONS_1	1
Known Hereditary Genetic Disorders		
POGONIS.DIAGNOSIS	PRED_CONDITIONS_2	1
Total Nephrectomy		
POGONIS.CANCER_SURG	SPECIFY	LEFT NEPHRECTOMY, LEFT NEPHRECTOMY AND ADRENALECTOMY, LEFT NEPHRECTOMY AND BX R KIDNEY LESION NEPHRECTOMY, NEPHRECTOMY & LYMPH NODE DISECTION, NEPHRECTOMY & PARTIAL RESECT INF. VENA CAVA NEPHRECTOMY (LT RADICAL), NEPHRECTOMY (RADICAL, RIGHT),<br>NEPHRECTOMY (RT RADICAL), NEPHRECTOMY AND ADRENALECTOMY, NEPHRECTOMY AND TUMOUR THROMBECTOMY, NEPHRECTOMY BILATERAL,<br>NEPHRECTOMY LEFT, NEPHRECTOMY LEFT RADICAL, NEPHRECTOMY LEFT, SPLENECTOMY, OMENTUMECTOMY NEPHRECTOMY RIGHT<br>NEPHRECTOMY RIGHT RADICAL, NEPHRECTOMY, COMPLETE, NEPHRECTOMY, RIGHT, NEPHRECTOMY- LEFT, NEPHRECTOMY-BILATERAL&TRANSPLANT KIDNEY, NEPHRECTOMY-LEFT, NEPHRECTOMY-RT KIDNEY, NEPHRECTOMY-RT RADICAL, NEPHRECTOMY/ADRENOLECTOMY,<br>NEPHRECTOMY; UNILATERAL LAPAROSCOPIC LEFT<br>BILATERAL NEPHRECTOMY, LAPAROTOMY, RESECTION OF ABDO NBL AND RIGHT NEPHRE, LEFT OPEN RADICAL NEPHRECTOMY, LEFT RADIAL NEPHRECTOMY, LEFT RADICAL NEPHRECTOMY AND LYMPHADENECTOMY, LEFT SIDED LAPAROSCOPIC ASSISTED PARTIAL

© 2025 Lebel A et al. *JAMA Network Open*

		NEPHRECTO, LEFT SIDED RADICAL NEPHRECTOMY AND RETROPERITONEAL, LEFT, OPEN, RADICAL NEPHRECTOMY WITH LYMPH NODE DI, LT NEPHRECTOMY, LT RADICAL NEPHRECTOMY & LYMPH NODE DISSECTION, LT RADICAL NEPHRECTOMY, LT RADICAL NEPHRECTOMY OF THE LYMPH NODE, LT. RADICAL NEPHRECTOMY, Nephrectomy, RADICAL NEPHRECTOMY, PORT IN, APPENDECTOMY RIGHT NEPHRECTOMY, RIGHT NEPHROURETERECTOMY, RIGHT RADICAL NEPHRECTOMY, RIGHT RADICAL NEPHRECTOMY, REMOVAL OF RUPTURED TUM, RIGHT RADICAL NEPHROURETERECTOMY, RT NEPHRECTOMY, RT NEPHRECTOMY AND LT PARTIAL, RT NEPHRECTOMY AND LYMPH NODE DISSECTION, RT RADICAL NEPHRECTOMY, RT. NEPHRECTOMY, RT. RADICAL NEPHRECTOMY, TOTAL RIGHT NEPHRECTOMY
CIHI-DAD	CCP	6740, 6741, 6742, 6744
	CCI	1PC89DA, 1PC89LB, 1PC89PF, 1PC89QF, 1PC91AB, 1PC91DA, 1PC91LB, 1PC91PF, 1PC91QF, 1PD89DA, 1PD89LB, 1PD89PF, 1PD89QF
OHIP	Fee code	S413, S415, S416, S420, S421
Partial Nephrectomy		
POGONIS.CANCER_SURG	SPECIFY	BILATERAL PARTIAL NEPHRECTOMY, LAPAROSCOPIC-ASSISTED LEFT PARITAL NEPHRECTOMY, LAPAROSCOPIC-ASSISTED RIGHT OPEN PARTIAL NEPHRECTO, LT LOWER POLE PARTIAL NEPHRECTOMY, LT PARTIAL NEPHRECTOMY, PARTIAL NEPHRECTOMY, PARTIAL NEPHRECTOMY – RIGHT, PARTIAL NEPHRECTOMY ADRENALECTOMY, PARTIAL NEPHRECTOMY AND NEUROBLASTOMA RESECTION, PARTIAL NEPHRECTOMY LEFT, PARTIAL NEPHRECTOMY RIGHT, PARTIAL NEPHRECTOMY-BILATERAL, R SUBTOTAL NEPHRECTOMY, L KIDNEY EXCISIONAL BX S, RT PARTIAL NEPHRECTOMY
CIHI-DAD	CCP	673
	CCI	1PC87DA, 1PC87LA, 1PC87LAXXE, 1PC87LAXXG, 1PC87NQ

© 2025 Lebel A et al. *JAMA Network Open*

OHIP	Fee code	S411, S423
Abdominal Radiation		
POGONIS.RADIATION	SITE	ABDOMEN AND MEDIASTINUM, BILAT LUNG / LT ABDO, HEMI-ABDOMEN, LOWER ABDOMEN, OTHER-WHOLE ABDOMEN, PELVIS + R ABDO,<br>WHOLE ABDOMEN, WHOLE ABDOMEN AND PELVIS,<br>WHOLE ABDOMEN/PELVIS, WHOLE ABDOMEN,<br>abdomen – left, abdomen – right, hemi-abdomen,<br>whole abdomen
	SITE_SPECIFY	ABD NODES, ABD/PELVIS, ABDO/LT RENAL BED,<br>ABDO/MEDIAST/CLAVI, ABDO/RT RENAL BED,<br>ABDO/SPLEEN, ABDOMEN & THORAX,<br>ABDOMEN AND MANTLE, ABDOMEN NOS,<br>ABDOMEN/SPLEEN, ANT&POST ABDOMEN,<br>ANT. + POST. ABDO, ANTERIOR ABDOMEN,<br>ANTERIOR RIGHT ABDOMEN, AP and PA abdomen, Phase 1,<br>Abdomen (right) and pa nodes,<br>Abdomen + pelvis, Abdomen inverted Y,<br>Abdomen whole, abdomen right,<br>Abdomen-right/Lumbar spine,<br>Abdomen-whole, Pelvis, Abdomen/Pelvis,<br>CHEST/ABDO/LTHUME, L ABDO AND R HILIUM, L NECK, MIDDLE ABDO, L THORAX+ MID ABDOM,<br>LEFT UPPER ABDOMEN, LOWER ABDOMEN,<br>Lower Abdomen,<br>Lt.Hemi Abdomen,Para Aortic region, both Lungs,<br>Lung - bilateral + Abdomen – left,<br>Lung-bilateral, abdomen-whole,<br>Lung-right, Lung-Left, Abdomen Left,<br>MEDIAST. AND ABDOME, MOD MANTLE ABC+ABD,<br>Mantle, Upper Abdomen,

© 2025 Lebel A et al. *JAMA Network Open*

		Mantle, Upper abdomen / Spleen,<br>Mantle, upper abdomen & spleen,<br>Mantle/abdo., Mediastinum,Lung-right,Lung, left,Abdomen left, Neck-right, Abdomen-right,<br>PA NODES/ABDO, PART ABDOMEN T11-L2, PARTIAL ABDOMEN, PELVIS AND ABDOMEN, PELVISABDOLYMPH NO,POST. ABDO + SPLEEN, Paraaortic & abdomen,R ABDO, R ILIAC, FEMUR, R HEMITHORAX + ABDO, RIGHT UPPER ABDOMEN, EXCLUDING LEFT KIDNEY, RPO UPPER ABDOMEN, RT ABDOMEN, RT UPPER ABDOMEN, Right rib, Abdomen, Rt Rib, Abdomen, SKULL, FEMUR, ABD,SPLEEN + UPPER ABDO, THORACO-ABDOMINAL,UP. ABDOMEN/SPLEEN,UPPER ABDOMEN, Upper Abdomen, Upper abdomen, Upper abdomen & chest, WHOLE ABDO&PELVIS,<br>WHOLE ABDO, PELVIS, Whole abdomen, Pelvis, Abdomen, abdomen-left, lumbar spine, abdomen-left, orbit-right,abdominal, ant/post para-aortic nodes + rt. Abd, anterior, posterior abdomen,<br>chest & rt abdomen, lung-bilateral/abdomen-right,<br>mantle + upper abdomen to L2, mantle and inverted Y of abdomen,mantle, abdomen, pelvis, mantle/upper abdomen, mantle/upper abdomen/spleen, right abdomen and whole lung, right hemi-abdomen; left periorbital,<br>upper abdomen, upper mantle and upper abdomen,<br>whole abdomen and chest, whole abdomen ant./post - ant/post thorax, whole abdomen, pelvis, whole abdomen/pelvis
Renal Radiation		
POGONIS.RADIATION	SITE	FLANK ABDOMEN LEFT, FLANK/ABDOMEN RIGHT, FLANK/ABDOMEN RIGHT/LEFT/PELVIS
	SITE_SPECIFY	AP, PA, LOA and kidney, KIDNEY/PERINEPHRIC,<br>Kidney area and Spine, R KIDNEY BED,<br>pelvis, left kidney, (L) Renal bed, ABDO/LT RENAL BED, ABDO/RT RENAL BED, ANT&POST RENAL BED,

© 2025 Lebel A et al. *JAMA Network Open*

		ANT/POST RENAL BED, LEFT RENAL BED, LT ILIAC & RENAL VEINS,<br>PARA AORTIC/RENAL, RENAL VESSELS,<br>RIGHT RENAL BED, RT RENAL BED,<br>anterior/posterior renal beds,<br>left retroperitoneum, left renal hilus and paraort,<br>left pararenal anterior and posterior, renal bed,<br>renal fossa, renal fossa and paraortic region, renal fossa,<br>anterior/posterior, ABDO/LT FLANK, ABDOMEN/LEFT FLANK,<br>ABDOMEN/FLANK-LEFT,<br>whole lung, whole abdomen and right flank
Total Body Radiation		
POGONIS.RADIATION	SITE	total body irradiation (TBI)
	SITE_SPECIFY	TOTAL BODY, lung-bilateral, total body irradiation (TBI)
POGONIS.BMT	BMT_TYPE	AUTOLOGOUS, Autologous
Allogenic Stem Cell Transplant		
POGONIS.BMT	BMT_TYPE	ALLOGENEIC, Allogeneic
Exposure to Cisplatin		
POGONIS.CHEMO_AGENTS	DRUG_NAME	'*CISPLATIN'
Exposure to Carboplatin		
POGONIS.CHEMO_AGENTS	DRUG_NAME	' 'CARBOPLATINUM'
Exposure to Ifosfamide		
POGONIS.CHEMO_AGENTS	DRUG_NAME	*ISOPHOSPHAMIDE
Exposure to Methotrexate		
POGONIS.CHEMO_AGENTS	DRUG_NAME	'*METHOTREXATE', 'METHOTREXATE <1G', 'METHOTREXATE'
Exposure to Cyclophosphamide		
POGONIS.CHEMO_AGENTS	DRUG_NAME	' '*CYCLOPHOSPHAMIDE'
Outcomes		
Chronic Kidney Disease (including chronic dialysis and renal transplant)		
CIHI-DAD, SDS, NACRS	ICD9	585, 586, 7944, 2504, 4030, 4031,4039, 4040, 4041, 4049, 5888, 5889, V420, T861

© 2025 Lebel A et al. *JAMA Network Open*

	ICD10	N03, N11, E102, E112, E132, E142, I12, I13, N08, N18, N19, R944
	CCP	6759, 6743, 6750, 5195, 6698
	CCI	1PC85, 1PZ21
OHIP	Diagnostic code	403, 585
	Fee code	E762, E769, E771, G347, G348, G408, G409, G412, S434, S435, Z631, R849, G323, G325, G326, G860, G862, G865, G863, G866, G330, G331, G333, G861, G082, G083, G085, G090, G091, G092, G093, G094, G095, G096, G294, G295, G864, H540, H740
CORR.RECIPIENT_TREATMENT	TREATMENT_CODE	*Renal Transplant*<br>171<br><br>*Chronic Dialysis*<br>Not in 171 or 181
	TRANSPLANTED_ORGAN_TYPE_CODE[1-3]	10, 11, 12, 18, 18, 19
Hypertension		
CIHI-DAD, SDS, NACRS	ICD9	401, 402, 403, 404, 405, 4372, 4030, 4031, 4039, 4040, 4041,4049
	ICD10	I10, I11, I12, I13, I15, I674
OHIP	Diagnostic code	401, 402, 403
Renal Replacement Therapy		
CORR.RECIPIENT_TREATMENT	TREATMENT_CODE	*Renal Transplant*<br>171<br><br>*Chronic Dialysis*<br>Not in 171 or 181
	TRANSPLANTED_ORGAN_TYPE_CODE[1-3]	10, 11, 12, 18, 18, 19
CIHI-DAD, SDS, NACRS	ICD9	V420
	ICD10	T861
	CCP	6759, 6743, 6750, 5195, 6698
	CCI	1PC85, 1PZ21

© 2025 Lebel A et al. *JAMA Network Open*

OHIP	Fee code	E762, E769, E771, G347, G348, G408, G409, G412, S434, S435, Z631, R849, G323, G325, G326, G860, G862, G865, G863, G866, G330, G331, G333, G861, G082, G083, G085, G090, G091, G092, G093, G094, G095, G096, G294, G295, G864, H540, H740
Censoring Events		
Date of Last Contact+3 years		
CIHI-DAD, SDS	ADMDATE	
NACRS	REGDATE	
IPDB	MAINSPECIALTY	
OHIP	SPEC	
New Cancer Diagnosis or Relapse		
POGONIS.DIAGNOSIS	DIAGDATE	
POGONIS.DIAG_RELAPSE	RELAPSE_DATE	
OCR.DIAGNOSIS.INCIDENT_CASES2018	DXDATE	
Death		
RPDB	DTHDATE	

Abbreviations: CIHI: Canadian Institutes for Health Information, CORR: Canadian Organ Replacement Registry; DAD: Discharge Abstract Database; MOMBABY: Linked Delivering Mother and Newborns; NACRS: National Ambulatory Care Reporting System; OCR: Ontario Cancer Registry; OHIP: Ontario Health Insurance Plan, POGONIS: Pediatric Oncology Group of Ontario Networked Information

© 2025 Lebel A et al. *JAMA Network Open*

eTable 3. Cohort characteristics of CCS and two comparator cohorts (before matching)

Characteristic	CCS cohort N= 10,182	Hosp. cohort N= 831,214	Stand. Diff (%)[a]	GP cohort N= 2,145,854	Stand. Diff (%)[a]
**Demographics**					
**Age at diagnosis**					
Median (IQR) y	8 (4-13)	6 (2-15)	17*	8 (4-13)	1
≤1y- N (%)	1,048 (10.3%)	197,320 (23.7%)	36*	174,749 (8.1%)	7
>1y and ≤12y- N (%)	5,620 (55.2%)	335,907 (40.4%)	30*	1,307,077 (60.9%)	12*
>12y- N (%)	3,514 (34.5%)	297,987 (35.8%)	3	664,028 (30.9%)	8
**Age at index date**					
Median (IQR) y	7 (3-13)	5 (0-14)	19*	7 (3-12)	1
≤1y- N (%)	1,649 (16.2%)	303,173 (36.5%)	47*	318,808 (14.9%)	4
>1y and ≤12y- N (%)	5,514 (54.1%)	251,443 (30.3%)	50*	1,281,886 (59.7%)	11*
>12y- N (%)	3,019 (29.7%)	276,598 (33.3%)	8	545,160 (25.4%)	10*
**Male sex**- N (%)	5,529 (54.3%)	420,431 (50.6%)	7	1,095,310 (51.0%)	7
**Rural status**[b]- N (%)	1,103 (10.8%)	121,296 (14.6%)	11*	189,341 (8.8%)	7
**Income quantile**[c]					
1	1,833 (18.0%)	195,036 (23.5%)	14*	457,657 (21.3%)	8
2	1,869 (18.4%)	164,884 (19.8%)	4	408,621 (19.0%)	2
3	2,084 (20.5%)	169,222 (20.4%)	0	431,981 (20.1%)	1
4	2,231 (21.9%)	160,273 (19.3%)	6	433,179 (20.2%)	4
5	2,165 (21.3%)	141,799 (17.1%)	11*	414,416 (19.3%)	5
**Era**					
1993-2001	2,655 (26.1%)	357,049 (43.0%)	36*	210,420 (9.8%)	43*
2002-2010	3,495 (34.3%)	257,904 (31.0%)	7	738,139 (34.4%)	0
2011-2020	4,032 (39.6%)	216,261 (26.0%)	29*	1,197,295 (55.8%)	33*
**Comorbidities**[d]					
**Cardiac disease**	652 (6.4%)	39,450 (4.7%)	7	11,119 (0.5%)	33*
**Chronic liver disease**	261 (2.6%)	5,645 (0.7%)	15*	2,392 (0.1%)	21*
**Diabetes**	124 (1.2%)	13,997 (1.7%)	4	3,031 (0.1%)	13*
**PMCA**[e]					
Non-chronic	7,178 (70.5%)	772,542 (92.9%)	61*	2,131,205 (99.3%)	88*
Non-complex chronic	452 (4.4%)	37,877 (4.6%)	1	11,322 (0.5%)	25*
Complex chronic	2,552 (25.1%)	20,795 (2.5%)	69*	3,327 (0.2%)	81*
**Health care utilization**[f]					
**Hospitalizations**					
0	8,488 (83.4%)	649,204 (78.1%)	13*	2,033,429 (94.8%)	37*
1	1,269 (12.5%)	160,649 (19.3%)	19*	107,021 (5.0%)	27*
≥2	425 (4.2%)	21,361 (2.6%)	9	5,404 (0.3%)	27*
**ED visits**					
0	7,263 (71.3%)	662,581 (79.7%)	20*	1,835,299 (85.5%)	35*
1	1,642 (16.1%)	93,006 (11.2%)	14*	217,509 (10.1%)	18*
≥2	1,277 (12.6%)	75,627 (9.1%)	11*	93,046 (4.3%)	30*
**Primary care visits**					

© 2025 Lebel A et al. *JAMA Network Open*

0	1,299 (12.8%)	128,377 (15.4%)	8	877,844 (40.9%)	67*
1	1,043 (10.2%)	82,912 (10.0%)	1	291,814 (13.6%)	10*
≥2	7,840 (77.0%)	619,925 (74.6%)	6	976,196 (45.5%)	68*
**Specialist visits**					
0	7,595 (74.6%)	691,672 (83.2%)	21*	2,017,474 (94.0%)	55*
1	1,080 (10.6%)	70,632 (8.5%)	7	83,240 (3.9%)	26*
≥2	1,507 (14.8%)	68,910 (8.3%)	20*	45,140 (2.1%)	47*

Abbreviations: *CCS*: childhood cancer survivors; *ED*: emergency department; *GP*: general population; *Hosp.:* hospitalization; *IQR*: interquartile range; *PMCA*: pediatric medical complexity algorithm; *Stand.Diff*: standardized difference; *y*: years.

*Statistically significant (Stand.Diff ≥10%).

[a]Standardized difference were used to compare CCS with the hospitalization and with the GP cohorts (referent groups). A standardized difference ≥10% is considered a meaningful difference

[b]Rural status was defined as residence within a community <10,000 persons.

[c]Income quintile was defined as neighborhood income quintile by postal code.

[d]Comorbidities diagnosed in the year prior to cancer treatment start date in the CCS cohort, to hospitalization discharge date in the hospitalization cohort, and to a randomly assigned date (based on distribution of cancer treatment start dates for CCS cohort) in the GP cohort, were considered. Comorbidities diagnosed between cancer treatment start and end dates (or equivalent dates in the comparator cohorts as mentioned above) were also considered.

[e]PMCA classification is a validated algorithm, used to classify children with chronic disease according to medical complexity using administrative data.

[f]Health care utilization was evaluated 1 month to 13 months prior to cancer treatment start date in the CCS cohort, to hospitalization discharge date in the hospitalization cohort, and to a randomly assigned date (based on distribution of dates for CCS cohort) in the GP cohort.

© 2025 Lebel A et al. *JAMA Network Open*

eFigure: Cumulative incidence function curves for CKD, hypertension and CKD or hypertension (primary outcome) in CCS versus comparator cohorts.

a) CCS *vs.* Gen Pop: CKD or hypertension

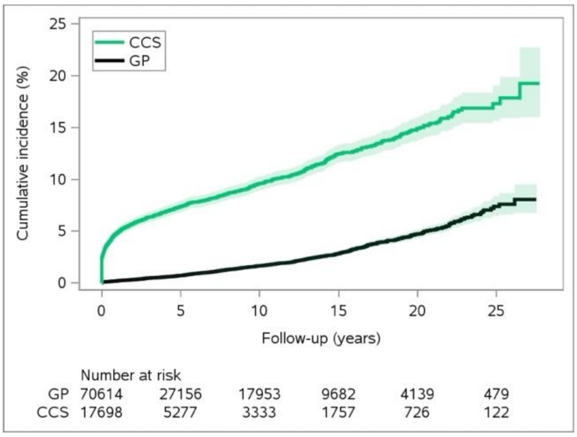

d) CCS *vs.* Hosp Cohort: CKD or hypertension

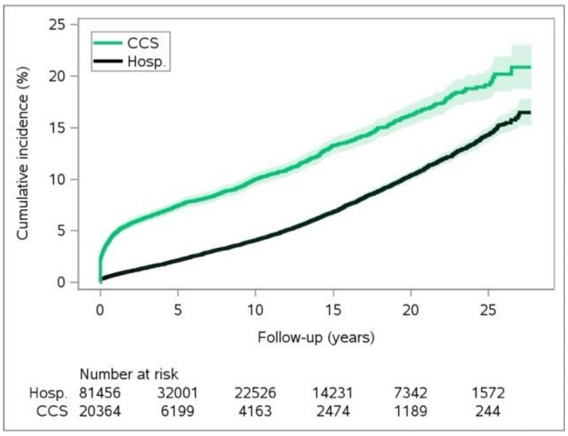

b) CCS *vs.* Gen Pop: CKD

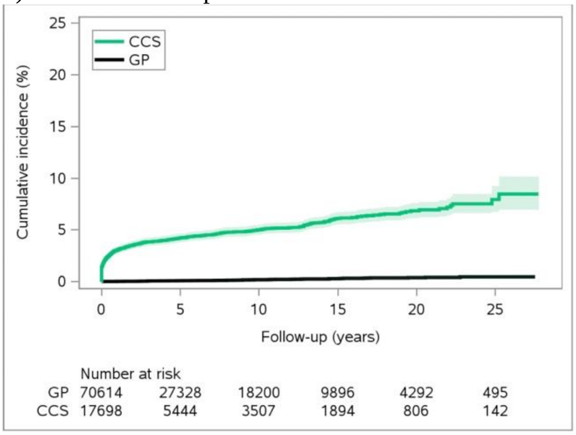

e) CCS *vs.* Hosp Cohort: CKD

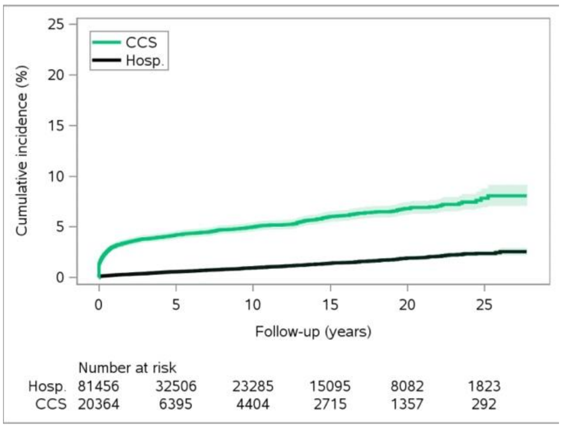

c) CCS *vs.* Gen Pop: hypertension

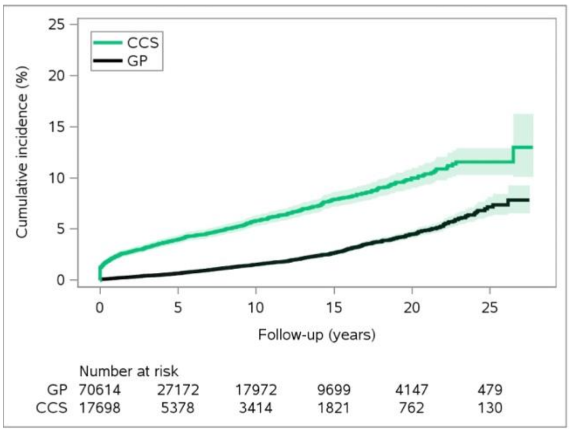

f) CCS *vs.* Hosp Cohort: hypertension

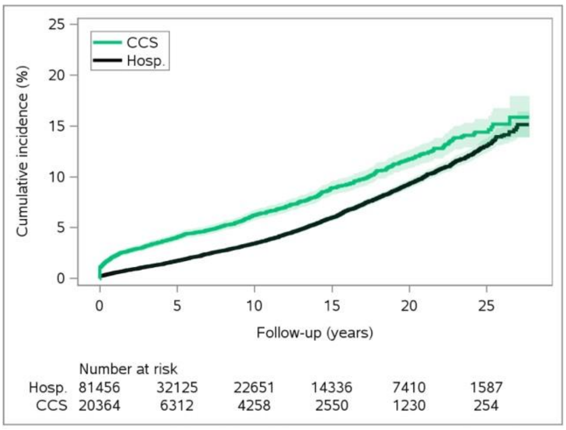

eFigure 1 Legend. All panels show the cumulative incidence function curves for the CCS and comparator cohorts for the development of the primary outcome (CKD or hypertension) and for CKD and hypertension individually. Number at risk refers to the number of individuals remaining available for observation over time. Shaded areas represent 95% confidence intervals for the curves.

Abbreviations: *CKD:* chronic kidney disease; *CCS:* childhood cancer survivors; *Gen Pop:* general population cohort; *GP:* General population cohort; *Hosp:* Hospitalization cohort.

© 2025 Lebel A et al. *JAMA Network Open*

eTable 4: Cancer characteristics of CCS

Characteristic	CCS cohort<br>N= 10,182
**Cancer type**- N (%)	
Leukemia, myeloproliferative diseases, and MDS	2,948 (29.0%)
CNS neoplasms	2,123 (20.9%)
Lymphoma and reticuloendothelial neoplasms	1,583 (15.5%)
Soft tissue and other extraosseous sarcomas	753 (7.4%)
Neuroblastoma and other peripheral nervous cell tumors	611 (6.0%)
Malignant bone tumors	453 (4.4%)
Wilm's tumor and other renal tumors	438 (4.3%)
Germ cell tumors and other neoplasms of gonads	432 (4.2%)
Other malignant epithelial neoplasms	425 (4.2%)
Retinoblastoma	240 (2.4%)
Hepatic tumors	107 (1.1%)
Other and unspecified malignant neoplasms	69 (0.7%)
**Treatment duration-** median (IQR) days	176 (90-712)
**Chromosomal abnormalities**- N (%)	293 (2.9%)
**Known hereditary genetic disorder**- N (%)	267 (2.6%)
**Total nephrectomy** - N (%)	369 (3.6%)
**Partial nephrectomy**- N (%)	87 (0.9%)
**Irradiation to kidneys area**- N (%)	80 (0.8%)
**Irradiation to abdomen**- N (%)	238 (2.3%)
**Total body irradiation**- N (%)	60 (0.6%)
**Autologous stem cell transplantation**- N (%)	297 (2.9%)
**Allogeneic stem cell transplantation**- N (%)	246 (2.4%)
**Any Nephrotoxic chemotherapy**- N (%)	4,978 (48.9%)
**Cisplatin therapy**- N (%)	1,064 (10.4%)
**Cisplatin dose**[a]- median (IQR) mg/m^2	400 (240-480)
**Carboplatin therapy**- N (%)	875 (8.6%)
**Ifosfamide therapy**- N (%)	1,072 (10.5%)
**Ifosfamide dose**[a]- median (IQR) mg/m^2	24,000 (9,000-48,000)
**High-dose Methotrexate therapy**- N (%)	1,805 (17.7%)
**High-dose Methotrexate dose**[a]- median (IQR) mg/m^2	12,000 (6,000-20,000)
**Cyclophosphamide therapy**- N (%)	4,268 (41.9%)
**Cyclophosphamide dose**[a]- median (IQR) mg/m^2	3,000 (2,000-5,000)

Abbreviations: *CCS*: Childhood cancer survivors; *CNS*: central nervous system; *IQR*: interquartile range; *MDS*: myelodysplastic syndrome;
[a] Cumulative dose.

© 2025 Lebel A et al. *JAMA Network Open*

eTable 5: Analyses by demographic and clinical subgroups: adjusted hazard ratio for CKD or HTN in CCS compared with two comparator cohorts

		Hazard ratio for CKD or HTN, adjusted for cardiac disease, liver disease, diabetes			
**Subgroups**	**Cohort**	**aHR**	**95% CI**	**p-value**	**Interaction p-value**
**CCS cohort versus general population cohort subgroup analyses**					
**Sex**- female	GP	1.00 (referent)			0.80
	CCS	4.71	(4.05-5.46)	<0.001	
**Sex**- male	GP	1.00 (referent)			
	CCS	4.72	(4.14-5.38)	<0.001	
**Age** ≤1y	GP	1.00 (referent)			0.12
	CCS	5.15	(2.92-9.08)	<0.001	
**Age** >1y and ≤12y	GP	1.00 (referent)			
	CCS	4.82	(4.29-5.42)	<0.001	
**Age** >12y	GP	1.00 (referent)			
	CCS	4.34	(3.59-5.26)	<0.001	
**Cancer type**- Leukemias, myeloproliferative diseases, and myelodysplastic diseases	GP	1.00 (referent)			<0.001
	CCS	5.77	(4.90, 6.79)	<0.001	
**Cancer type**- Lymphomas and reticuloendothelial neoplasms	GP	1.00 (referent)			
	CCS	3.36	(2.49, 4.52)	<0.001	
**Cancer type-** CNS and miscellaneous intracranial and intraspinal neoplasms	GP	1.00 (referent)			
	CCS	2.70	(2.14, 3.42)	<0.001	
**Cancer type-** Other Cancers	GP	1.00 (referent)			
	CCS	5.74	(4.83, 6.81)	<0.001	
**Stem cell transplant -** No	GP	1.00 (referent)			<0.001
	CCS	4.45	(4.01, 4.92)	<0.001	
**Stem cell transplant -** Yes	GP	1.00 (referent)			
	CCS	9.91	(6.60, 14.87)	<0.001	
**Cisplatin use-** No	GP	1.00 (referent)			0.03
	CCS	4.55	(4.09, 5.05)	<0.001	
**Cisplatin use-** Yes	GP	1.00 (referent)			
	CCS	6.14	(4.58, 8.24)	<0.001	
**Ifosfamide use-** No	GP	1.00 (referent)			0.15
	CCS	4.59	(4.13, 5.10)	<0.001	
**Ifosfamide use-** Yes	GP	1.00 (referent)			
	CCS	5.50	(4.18, 7.24)	<0.001	
**High dose methotrexate use -** No	GP	1.00 (referent)			0.14
	CCS	4.50	(4.02, 5.04)	<0.001	
**High dose methotrexate use -** Yes	GP	1.00 (referent)			
	CCS	5.42	(4.42, 6.64)	<0.001	
**Nephrectomy-** No	GP	1.00 (referent)			<0.001
	CCS	4.41	(3.98, 4.89)	<0.001	
**Nephrectomy-** Yes	GP	1.00 (referent)			
	CCS	11.42	(7.72, 16.88)	<0.001	

© 2025 Lebel A et al. *JAMA Network Open*

**Radiation use-** No	GP	1.00 (referent)			0.005
	CCS	4.55	(4.11, 5.03)	<0.001	
**Radiation use-** Yes	GP	1.00 (referent)			
	CCS	8.30	(5.41, 12.76)	<0.001	
**Era** 1993-2001	GP	1.00 (referent)			<0.001
	CCS	3.49	(2.91, 4.19)	<0.001	
**Era** 2002-2010	GP	1.00 (referent)			
	CCS	3.92	(3.36, 4.58)	<0.001	
**Era** 2011-2020	GP	1.00 (referent)			
	CCS	8.15	(6.75, 9.84)	<0.001	
**CCS cohort versus hospitalization cohort subgroup analyses**					
**Sex**- female	Hospitalization	1.00 (referent)			0.05
	CCS	1.84	(1.66-2.04)	<0.001	
**Sex**- male	Hospitalization	1.00 (referent)			
	CCS	2.14	(1.94-2.35)	<0.001	
Age ≤1y	Hospitalization	1.00 (referent)			<0.001
	CCS	2.43	(1.58-3.71)	<0.001	
Age >1y and ≤12y	Hospitalization	1.00 (referent)			
	CCS	2.45	(2.23-2.69)	<0.001	
Age >12y	Hospitalization	1.00 (referent)			
	CCS	1.53	(1.37-1.70)	<0.001	
**Cancer type**- Lymphomas and reticuloendothelial neoplasms	Hospitalization	1.00 (referent)			<0.001
	CCS	2.76	(2.45, 3.12)	<0.001	
**Cancer type**- Lymphomas and reticuloendothelial neoplasms	Hospitalization	1.00 (referent)			
	CCS	1.38	(1.15, 1.66)	<0.001	
**Cancer type-** CNS and miscellaneous intracranial and intraspinal neoplasms	Hospitalization	1.00 (referent)			
	CCS	1.35	(1.13, 1.61)	0.001	
**Cancer type-** Other Cancers	Hospitalization	1.00 (referent)			
	CCS	5.74	(4.83, 6.81)	<0.001	
**Stem cell transplant -** No	Hospitalization	1.00 (referent)			<0.001
	CCS	1.87	(1.74, 2.01)	<0.001	
**Stem cell transplant -** Yes	Hospitalization	1.00 (referent)			
	CCS	4.41	(3.41, 5.69)	<0.001	
**Cisplatin use-** No	Hospitalization	1.00 (referent)			0.001
	CCS	1.92	(1.78, 2.07)	<0.001	
**Cisplatin use-** Yes	Hospitalization	1.00 (referent)			
	CCS	2.61	(2.13, 3.20)	<0.001	
**Ifosfamide use-** No	Hospitalization	1.00 (referent)			0.52
	CCS	2.00	(1.85, 2.15)	<0.001	
**Ifosfamide use-** Yes	Hospitalization	1.00 (referent)			
	CCS	1.90	(1.57, 2.30)	<0.001	
**High dose methotrexate use -** No	Hospitalization	1.00 (referent)			<0.001
	CCS	1.85	(1.71, 2.00)	<0.001	
**High dose methotrexate use -** Yes	Hospitalization	1.00 (referent)			
	CCS	2.79	(2.39, 3.27)	<0.001	
**Nephrectomy-** No	Hospitalization	1.00 (referent)			

© 2025 Lebel A et al. *JAMA Network Open*

	CCS	1.89	(1.76, 2.03)	<0.001	<0.001
**Nephrectomy-** Yes	Hospitalization	1.00 (referent)			
	CCS	5.42	(4.10, 7.18)	<0.001	
**Radiation use-** No	Hospitalization	1.00 (referent)			<0.001
	CCS	1.93	(1.80, 2.08)	<0.001	
**Radiation use-** Yes	Hospitalization	1.00 (referent)			
	CCS	3.74	(2.76, 5.07)	<0.001	
**Era** 1993-2001	Hospitalization	1.00 (referent)			<0.001
	CCS	1.53	(1.37, 1.70)	<0.001	
**Era** 2002-2010	Hospitalization	1.00 (referent)			
	CCS	1.93	(1.71, 2.18)	<0.001	
**Era** 2011-2020	Hospitalization	1.00 (referent)			
	CCS	3.45	(2.97, 4.01)	<0.001	

Abbreviations: *aHR*: adjusted hazard ratio; *CCS*: childhood cancer survivors; *CI*: confidence interval; *CKD*: chronic kidney disease; *GP*: general population; *HTN*: hypertension; *y*: years.

© 2025 Lebel A et al. *JAMA Network Open*
