# Supplementary material for: Chronic Kidney Disease or Hypertension After Childhood Cancer
Source: JAMA Netw Open. 2025 May 19;8(5):e258199. doi: 10.1001/jamanetworkopen.2025.8199 (PMC12090035; doi:10.1001/jamanetworkopen.2025.8199)
Supplement: Supplement 2. — Data Sharing Statement [file jamanetwopen-e258199-s002.pdf]

Data Sharing Statement

Lebel. Chronic Kidney Disease or Hypertension After Childhood Cancer. *JAMA Netw Open*. Published May 01, 2025. doi:10.1001/jamanetworkopen.2025.8199

Data
Data available: No

Additional Information
Explanation for why data not available: As per regulations and ethical requirements for the use of administrative healthcare data in Ontario, Canada and by ICES, the data used for this study cannot and will not be shared. However, investigators may contact ICES to query how to access ICES data and analysts for future new or confirmatory studies (https://www.ices.on.ca/use-ices-data/). For transparency, the variable definitions and diagnoses used to identify our exposures and outcomes are provided in the supplementary information.
